# Interaction between IGFBP7 and insulin: a theoretical and experimental study

**DOI:** 10.1038/srep19586

**Published:** 2016-04-22

**Authors:** Wenjing Ruan, Zhengzhong Kang, Youzhao Li, Tianyang Sun, Lipei Wang, Lijun Liang, Maode Lai, Tao Wu

**Affiliations:** 1 Department of Pathology, School of Medicine, Zhejiang University, Hangzhou 310058, P. R. China; 2 Soft Matter Research Center and Department of Chemistry, Zhejiang University, Hangzhou 310027, P. R. China; 3 Department of Respiratory Medicine, Sir Run Run Shaw Hospital, School of Medicine, Zhejiang University, Hangzhou 310006, P.R. China

## Abstract

Insulin-like growth factor binding protein 7 (IGFBP7) can bind to insulin with high affinity which inhibits the early steps of insulin action. Lack of recognition mechanism impairs our understanding of insulin regulation before it binds to insulin receptor. Here we combine computational simulations with experimental methods to investigate the interaction between IGFBP7 and insulin. Molecular dynamics simulations indicated that His200 and Arg198 in IGFBP7 were key residues. Verified by experimental data, the interaction remained strong in single mutation systems R198E and H200F but became weak in double mutation system R198E-H200F relative to that in wild-type IGFBP7. The results and methods in present study could be adopted in future research of discovery of drugs by disrupting protein–protein interactions in insulin signaling. Nevertheless, the accuracy, reproducibility, and costs of free-energy calculation are still problems that need to be addressed before computational methods can become standard binding prediction tools in discovery pipelines.

Insulin is the most important and effective physiological anabolic agent released by pancreatic beta cells in response to elevated nutrient levels; it stimulates the storage of glucose and fatty acids, and inhibits muscle proteolysis^1^. Insulin resistance is a pathophysiological condition in which cells fail to respond to the normal actions of the hormone insulin. *In vivo*, beta cells in the pancreas subsequently increase their production of insulin, further contributing to hyperinsulinemia. Insulin resistance and hyperinsulinemia constitute the key abnormal pathophysiological state which leads to the pathogenesis of several diseases, including diabetes, metabolic syndrome, cancer, atherosclerosis, and stroke^2^,^3^. These chronic diseases have become the leading causes of mortality and disease worldwide. The estimated prevalence of diabetes in a representative sample of Chinese adults was 11.6% and the prevalence of prediabetes was 50.1%^4^.

When exploring the molecular mechanism of insulin resistance and hyperinsulinemia, considerable attention has been paid to the intracellular mechanisms of defective insulin signaling after bonding to the insulin receptor (IR). Some of these mechanisms involve the decreased expression and phosphorylation level of the phosphatidylinositol 3-kinase–AKT/protein kinase B pathways and the Ras–mitogen-activated protein kinase pathways after the autophosphorylation of IR and the tyrosine phosphorylation of insulin receptor substrates (IRS)^5^,^6^,^7^. To date, little is known about the regulation and mechanism of insulin in plasma before it binds to IR, which is as important as the intracellular signaling pathway. As an essential modulator of the bioavailability of insulin-like growth factors (IGFs) and insulin^8^, the secreted IGF binding protein 7 (IGFBP7) in human plasma plays roles in important diseases, such as cancer and diabetes^9^,^10^,^11^,^12^,^13^. The binding affinity of IGFBP7 to insulin is higher than other members of the IGFBP family. It decreases the phosphorylation level of the IR β subunit and IRS – namely, the early steps of insulin action - by blocking the binding of insulin to the IR^14^. However, the molecular mechanism behind the recognition of and interaction between IGFBP7 and insulin remains incompletely understood.

In this work, computational and experimental methods were used to investigate the interaction between IGFBP7 and insulin. The structure of IGFBP7 was determined by homology modeling. The interaction sites and key residues in the IGFBP7–insulin complex were predicted by docking, molecular dynamics (MD)/steered molecular dynamics (SMD) simulations, and free energy calculations. Hot-spot residues based on structural and chemical complementarity were identified and investigated through mutation experiments. Free-energy calculations based on umbrella sampling methods were carried out to determine the interaction energy between IGFBP7 and insulin.

The relationship between IGFBP7 and insulin investigated in this study will, we hope, go some way to understanding the regulation and mechanism of insulin in plasma, and the results of this study should impact on the design of drugs that regulate insulin and treat diseases related to insulin-mediated pathways.

## Results

### Homology modeling of IGFBP7 and prediction of hot-spot residues

**Figure 1:**
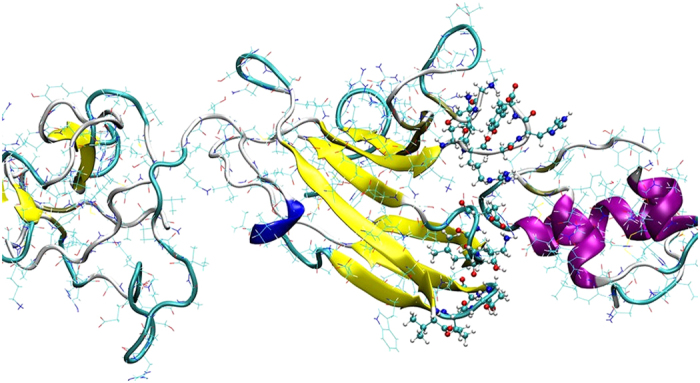
Recognition and interaction sites of IGFBP7 and insulin. Proteins were represented by New Cartoon models. IGFBP7 is displayed in yellow and blue while insulin is in purple. The residues of IGFBP7 on the recognize surface are shown in CPK models.

The structure of IGFBP7 was determined using ESyPred3D software^15^ by homology modeling because no complete crystal structure of IGFBP7 was found. Fibroblast growth factor 2 chain C (ID = 1cvs) was used as a template to produce the structure of IGFBP7, and the sequence identity was 21.8%^16^,^17^. The X-ray structure of insulin was obtained from a protein data bank (PDB Code 2C8R). The amino acid sequence of insulin includes two chains that consist of three alpha helices. Docking results and Visual Molecular Dynamics^18^ showed that the C-terminus of IGFBP7 was the principal recognition site of IGFBP7 and insulin (Fig. 1). This result is consistent with that of a previous study^14^.

Electrostatic interaction, particularly electrostatic complementarity, has a dominant role in protein–protein+ interactions (PPIs)^19^,^20^,^21^,^22^. Thus, we believe that the electrostatic potential of the hot-spot residues would vary significantly during the desorption process reconstructed from SMD simulation, reflecting the possible crucial role of electrostatic interactions in binding processes.

**Figure 2:**
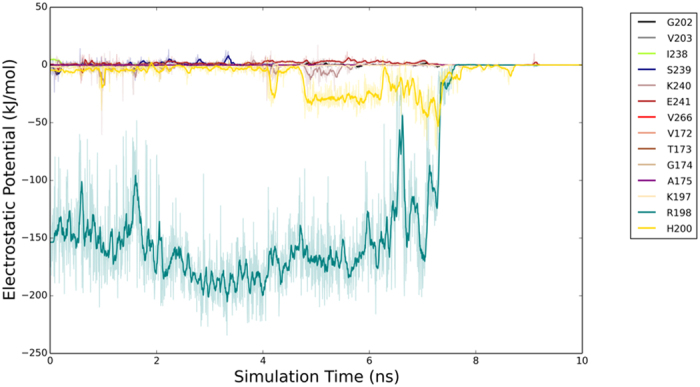
Electrostatic potential of each residue on the recognize surface of wild-type IGFBP7. Residues of IGFBP7 within 0.8 nm of insulin are chosen to calculate the electrostatic potential. H200 (in gold color) and R198 (in green color) stand out among the residues due to the trend of the potential curves.

The electrostatic potential of each residue on the contact surface of IGFBP7 was calculated from the SMD simulation. As shown in Fig. 2, the residues were divided into two types in terms of their trend of electrostatic potential variation.

The electrostatic potential of the two residues (Arg198 and His200) of IGFBP7 oscillated, increased, and stabilized during desorption process when insulin was pulled away from IGFBP7. By contrast, the electrostatic potential of the other residues of IGFBP7 are too small which can be neglected (Fig. 2).

The electrostatic potential results and SMD snapshots (not shown) indicated that His200 (Supplementary Fig. 1) and Arg198 strongly interacted with insulin. Hence, His200 and Arg198 were selected for the mutation experiments. The qualitative strength of interactions between IGFBP7 and insulin is shown in Table 1. His200 and Arg198 were found to be the key residues in the interaction between IGFBP7 and insulin. As shown in Table 1, the mutants of His200Phe and Arg198Ile exhibited very strong interaction. This result indicated that a single mutation cannot disrupt the interaction between IGFBP7 and insulin. The interaction between IGFBP7 and insulin was disrupted with the double mutations of His200 and Arg198. This result suggested that a few residues instead of only one residue need to be mutated to disrupt the interaction between IGFBP7 and insulin. PPIs are critically dependent on the hot-spot key residues at the interface^23^. In the present study, His200 and Arg198 were considered as these hot-spot residues - that is, these residues are dominant in their contribution to the binding energy.

### Mutation experiments of hot-spot residues

**Figure 3:**
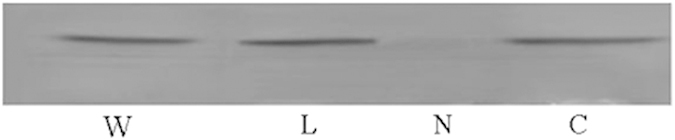
Binding of wild-type IGFBP7 and IGFBP7 fragments to insulin shown by western blot. Up to 20 μl insulin (1 μg/μl) was subjected to SDS–PAGE and then electroblotted on to nitrocellulose membranes. The membranes were then incubated with wild-type IGFBP7 or fragments. After washing, the membranes were incubated with anti-His antibody and then incubated in fluorescence-labeled secondary antibodies. The bands were detected with the imaging system and quantified. Lanes from left to right, wild-type IGFBP7, IGFBP7-L fragment (172–244), IGFBP7-N fragment (1–171), IGFBP7-C fragment (245–282).

**Figure 4:**
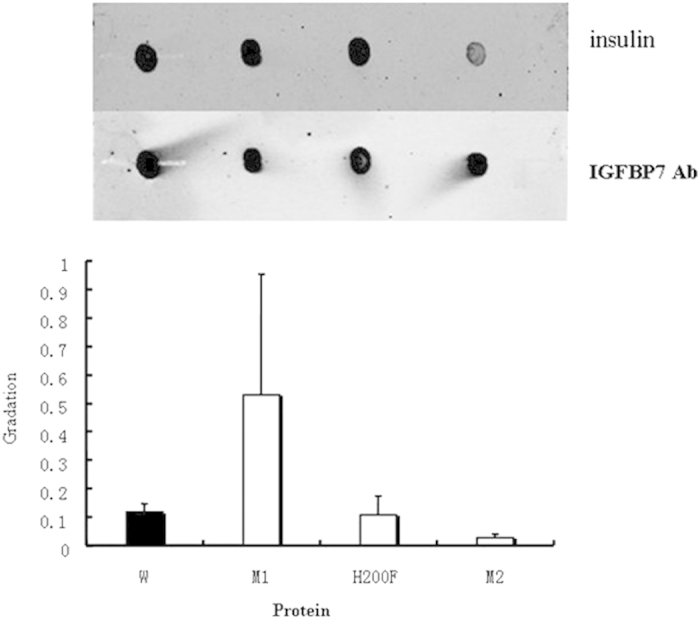
Ligand dot blot of IGFBP7 mutants with insulin and IGFBP7 antibody. 2.5 μl (0.09 mg/ml) wild-type IGFBP7 and IGFBP7 mutants were dotted directly on to the nitrocellulose membrane. After blocking, the membrane was incubated at 4 °C overnight with 1 μg/ml insulin. Membranes were washed and incubated with insulin antibody (upper panel). Lanes from left to right, wild-type IGFBP7, R198E mutant, H200F mutant, R198I-H200F mutant. Membranes were incubated with IGFBP7 antibody (lower panel), showing the presence of approximately equal amounts of protein. All blots shown are representative of at least three separate experiments, with error bars representing SD. The graph shows densitometric analysis of dots.

**Figure 5:**
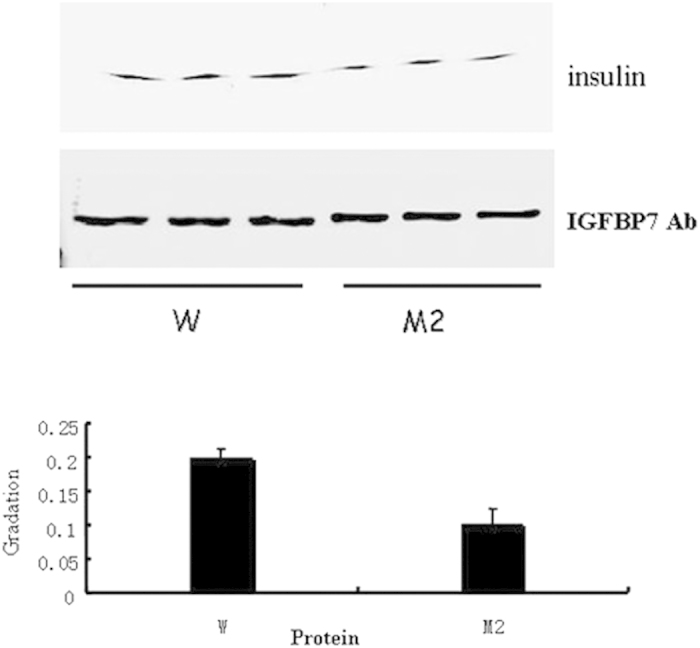
Pull-down assay of IGFBP7 mutants with insulin and IGFBP7 antibody. His6-tagged-IGFBP7 (0.1 mg, wild-type and mutants) were mixed with 0.5 mg insulin. The mixture was loaded on to a Ni-Sepharose 6 column. The eluted materials were subjected to SDS-PAGE and Western blotting with the anti–insulin antibody (upper panel) or the anti–IGFBP7 antibody (lower panel). Lanes from left to right, wild-type IGFBP7, R198I-H200F mutant, in triplicate. All bands shown are representative of at least three separate experiments, with error bars representing SD. The graph shows densitometric analysis of bands.

IGFBP7 fragments (N, L, C) and IGFBP7 mutants (R198E, R198I-H200F, and H200F) were constructed by PCR amplification and expressed as His-fusion cell lysates in *E. coli.* Amplification of the peptides was confirmed by SDS–PAGE and Coomassie staining. Binding of wild-type IGFBP7 and IGFBP7 fragments to insulin was performed by western blot analysis (Fig. 3). The binding bands of IGFBP7-N fragments to insulin were almost undetected, suggesting that the binding domain of IGFBP7 to insulin is located at the C-terminus, verifying our prediction. According to our mutants, sequencing analysis showed that Arg198 and His200 were mutated into various combinations: Arg198Glu (R198E), Arg198Ile and His200Phe (R198I-H200F), and His200Phe (H200F). Initial binding studies were performed by dot blot analysis (Fig. 4, upper panel). The binding ability to insulin was strengthened when Arg198 was changed to Glu. This finding is remarkably distinct compared with the qualitative strength result (Table 1). The binding ability to insulin was slightly changed when His200 was substituted by Phe. However, a clear reduction in binding was observed with the substitution of R198I together with the substitution of H200F. The weakened binding ability of the R198I-H200F mutant to insulin was also tested in a pull-down assay (Fig. 5). In this study, the binding ability to insulin clearly decreased when Arg198 and His200 were both mutated. This result is consistent with results obtained from computational methods.

### Molecular Mechanism by MD and binding free-energy calculation

PPIs are mainly contributed by van der Waals, electrostatic, and hydrogen bonding interactions, especially electrostatic interaction. The natural charge of the residues was changed in H200F and R198I, which led to the loss of electrostatic interaction between IGFBP7 and insulin. However, strong interaction was retained in the single mutation systems R198I and H200F, reflecting the mutational robustness of IGFBP7 in binding to insulin, as in many other interactions^24^,^25^.

In the present study, the R198I, H200F, and R198I-H200F systems were used to investigate the importance of Arg198 and His200, and compare the experimental and computational results in single mutation systems. PMF was successfully reconstructed through umbrella sampling method to capture the essence of protein recognition.

**Figure 6:**
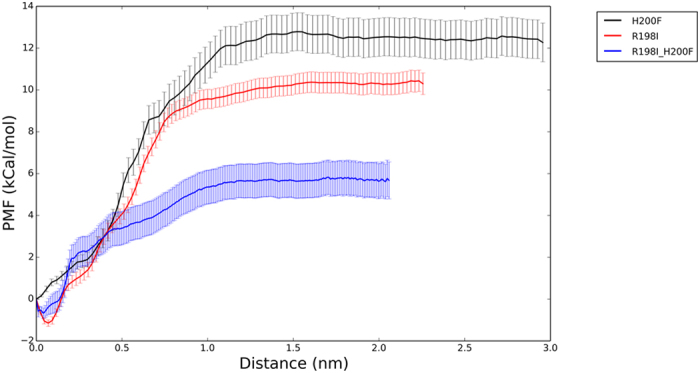
PMF along the distance change in different mutants. The distance change is calculated by initial distance between insulin and IGFBP7. Black and red lines represent H200F and R198I mutants, respectively. Blue line is for double mutants. Error bars are shown on each lines.

All PMF values in the mutants were positive, and the free energy of all mutants increased with distance change during the desorption process through SMD simulation as shown in Fig. 6.

These results indicated that the inverse process was spontaneous and that the interaction between IGFBP7 and insulin was more or less disrupted with mutation. H200F and R198I-H200F had the largest (12.5 ± 0.9 kCal/mol) and smallest (5.69 ± 0.8 kCal/mol) PMF values, respectively while the value of R198I mutant is 10.3 ± 0.5 kCal/mol which is quite close to that of H200F mutant. The process of SMD became complicated as the free energy was changed. Thus, the interaction between IGFBP7 and insulin was weaker in R198I-H200F than in R198I and H200F. The maximum disruption effect was observed in the mutation of R198I-H200F, which was consistent with the experiments. The results confirmed that Arg198 and His200 were the hot-spot residues in the binding between IGFBP7 and insulin, showing strong robustness.

**Figure 7:**
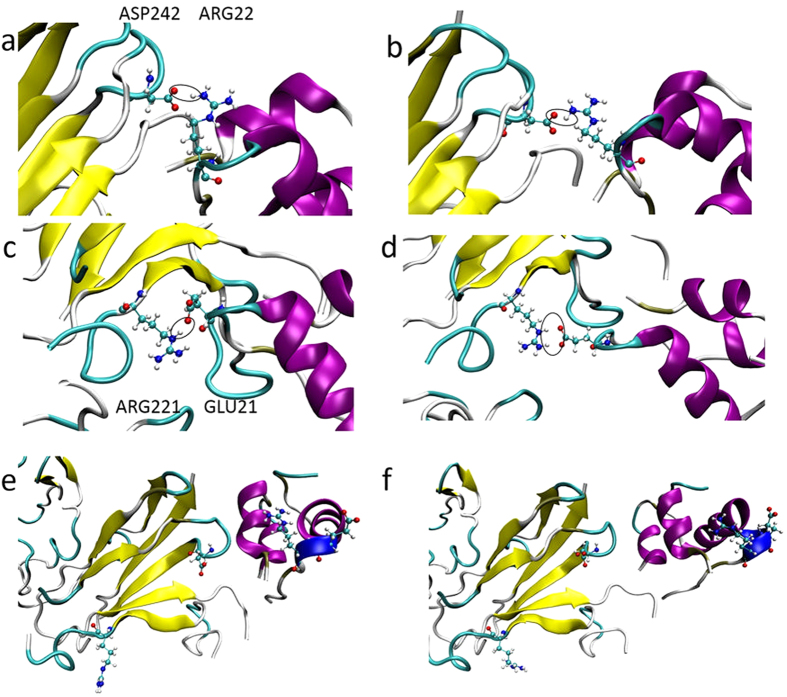
New bonding sites in mutant complex. Protein was represented by New Cartoon models. IGFBP7 and insulin are shown in yellow and purple, respectively. Side chains of Asp242, Arg22, Arg221 and Glu21 in the binding sites are represented by CPK model, coloring by atom type (C: cyan, N: blue, O: red, H: white). The hydrogen bond between Asp242 and Arg22, Arg221 and Glu21 are shown in black circle. Water molecules and ions are not displayed for clarity. (**a**–**f**) are snapshots for H200E, R198I and R198I-H200F, respectively. (**a**,**c**,**e**) are the initial snapshots while (**b**,**d**,**f**) are the snapshots at 4 ns when insulin was pulling away.

In SMD, the robust mechanism behind the recognition of IGFBP7 and insulin was clearly shown by the snapshots (Fig. 7). Although the interaction between hot-spot residues Arg198 and His200 was almost disrupted by mutation, the negative loop of IGFBP7 exhibited a key supporting function in the recognition of IGFBP7 mutants and insulin by rearranging the binding interface. Negative residues, Asp242 and Arg221, increased the mutational robustness of IGFBP7 binding to insulin, which did not show any contact or interaction signs from primary calculations (Fig. 2). As shown in the left part of Fig. 7, the Asp242 of IGFBP7 showed a strong interaction with the Arg22 and of insulin in H200F mutant (Fig. 7a) while Arg221 bond to Glu21 in R198I mutant (Fig. 7a). In the beginning of SMD, hydrogen bonds were formed between the Asp242 and Arg22 (Fig. 7a), Arg221 and Glu21 (Fig. 7c). As shown in Fig. 7d,f, the hydrogen bonds were completely disrupted in the double mutation system (R198I-H200F). However, it was well retained in the H200F and R198I system (Fig. 7b,d).

The interaction between IGFBP7 and insulin was principally contributed by the His200 and Arg198 of IGFBP7. When His200 or Arg198 was mutated into other residues, the negative loop of IGFBP7 acted as the key residue that interacted with insulin. Asp242/Arg221 were closer to Arg22/Glu21 in the single mutant system than in the double mutant system. The interaction between IGFBP7 and insulin was principally contributed by a small number of residues. When H200F or R198I was mutated, Asp242 and Arg221 acted as the key residues for maintaining a strong interaction with insulin. The single mutation complex of IGFBP7 and insulin showed a large conformational change, especially the side chain of the IGFBP7 mutants. The interaction mechanism of His200 and Arg198 with insulin was considered as cooperative and complementary. These results indicated that IGFBP7 relied on only a few key residues to interact with insulin. The robustness of IGFBP7 generally facilitated the recognition of insulin; however, other residues can also influence the interaction with insulin by triggering conformational changes during the mutation of key residues. These findings further prove that protein can be evolutionary in a conservative state. However, it should occur within the tolerance range of protein to prevent the disruption of PPIs, as observed in the double mutation system of IGFBP7. The Arg221 and Asp242 of IGFBP7 in the single mutation system were more flexible than those in the double mutation system, which could also be reflected by trajectory animation. The double mutation system of IGFBP7 was more rigid with the negative loop limited closely to Arg22 and Glu21. Similar to previous studies^26^, the present study showed through simulations that the flexibility of proteins is an important component in predicting protein–protein binding.

## Discussion

Our results reveal a robust mechanism of IGFBP7–insulin recognition at the molecular level. Preliminary investigations using docking and SMD simulations may neglect important details of PPIs. Many cellular functions rely on interactions among proteins. Disruptions in protein interactions are avoided by evolutionarily designing sequences to fold efficiently and robustly to a unique structure and to bind specifically and selectively^27^. Deeds *et al.*^28^. Found that a certain degree of evolutionary design of specific PPI strength is necessary to avoid the loss of specific protein complexes to possible promiscuous interactions with other proteins in the cell. That is, PPIs rely on a few key residues because of their robustness. The present study confirmed through molecular simulation and experiments that His200 and Arg198 were the hot-spot residues. As shown in Table 1, the single mutation systems H200F and R198I cannot disrupt the interaction between IGFBP7 and insulin. Experiments and free-energy calculations also showed that R198I mutation cannot disrupt this interaction. In accordance with previous studies^28^, the present study proved that PPIs are robust. The IGFBP7 mutants still interacted with insulin except when two key residues of IGFBP7 were both mutated, indicating the robustness of IGFBP7. This robustness seems to be an evolutionary adaptation to reduce the effect of mutations. Therefore, IGFBP7 can be effectively used to recognize insulin.

Complementarity patterns are important determinants in protein recognition and interaction between protein complexes, which are mainly driven by non-covalent interactions^29^,^30^. The hot-spot residues His200 and Arg198 were found to have important functions in the interaction between IGFBP7 and insulin. These residues help IGFBP7 recognize and bond to insulin, and these processes are enhanced by the robustness of IGFBP7. The physicochemical complementarities and robustness of protein would strengthen the bond between IGFBP7 and insulin, leading to the formation of a stable protein complex.

The molecular recognition of insulin and IGFBP7 represents a mutational robustness mechanism. Experimental and computational methods, including electrostatic potential, free-energy calculation, and molecular dynamics simulation, facilitate a deeper understanding of the mechanisms behind the intracellular or extracellular regulation of insulin. These methods can be applied to predict the complex formed between interacting proteins.

Insulin resistance and hyperinsulinemia are common pathophysiological features of some chronic diseases, such as diabetes, cardiovascular diseases, and cancer; abrogating insulin resistance will be a promising therapy targeting these diseases^31^,^32^. IGFBP7 binds to insulin and thereafter inhibits the binding of insulin to IR, impairing the subsequent signaling, which at least in part leads to the pathological state of insulin resistance and hyperinsulinemia. Based on our present finding on the binding sites of IGFBP7 to insulin, future study may focus on the small peptide design disrupting the IGFBP7-insulin interaction. We hypothesized that the disruption of IGFBP7 to insulin will be capable of restoring the dysfunction of insulin. The “insulin resistance” state will thus be partially relieved. The hypothesis seems attractive in the treatment of diseases involving insulin resistance.

As a generic property of many complex networks, robustness, the tolerance against errors, prevents the loss of connectivity of networks when facing key component malfunction or local failure^33^,^34^. If we introduce the network idea in insulin biological activities we may regard the insulin metabolism as a network and the interaction between insulin and IGFBP7 will be a linkage of the insulin network. The linkage study helps to uncover the direct interactions and correlations in the networks^35^,^36^. The insulin metabolism can be controlled or interrupted by several important insulin-bonding proteins reactions which is similar to the network that the structural controllability of the total network can be realized through a few driver nodes^37^ or even a single control input^38^. However we do not know whether linkages have the property of robustness just like that of the whole network and whether the system based on the stability provided by linkages rather than networks existence. Therefore, we chose the recognition issue between IGFBP7 and insulin, a linkage of insulin metabolic networks, as our studies. According to the computational and experimental data, the answers to these questions are positive in the insulin biological network system. The contribution of different linkages (insulin-protein interactions) to the network may differ from each other. Linkages with capability of robustness provide stability themselves, while linkages containing potential key driver nodes will guarantee that the networks are regulated by control inputs. In conclusion, the key linkages prediction is very important and special interaction characteristics should be considered when building mathematical models. It is worthy of study as to whether robustness is a good indicator of linkages prediction.

The methods used in the present study could be adopted in future research to focus on the discovery of drugs by disrupting PPIs in insulin signaling, especially for proteins without crystal structure such as G protein-coupled receptors. These approaches may be used as a guide for drug design in insulin- or IGF-mediated pathways. Nevertheless, the accuracy, reproducibility, and costs of free-energy calculation are still problems that need to be addressed before computational methods can become standard binding prediction tools in discovery pipelines.

## Methods

### Generation and purification of wild-type IGFBP7, IGFBP7 fragments and IGFBP7 mutants

An expression vector containing full-length human IGFBP7 cDNA was prepared as previously described^11^. According to our prediction, the binding domain of IGFBP7 and insulin resides in four amino acid regions: 172–176, 196–201, 217–220 and 235–244. The cDNA fragments of N (1–171), L (172–244), and C (245–282) were generated by polymerase chain reaction (PCR) amplification. The IGFBP7 mutants were constructed by substitution of residues Arg198 and His200. The cDNA fragments of the IGFBP7 mutants were generated by PCR amplification from human IGFBP7 cDNA. Mutations were introduced using the QuikChange^®^ Site-Directed Mutagenesis Kit (Stratagene). After sequencing sense and antisense strands, the fragments and the mutants were subcloned into pET28-a (His-tagged) and transformed into BL21DE3 *Escherichia coli* cells, cultured overnight in Luria–Bertani broth/kanamycin, and then induced with 1 mM isopropylthio-β-d-galactoside. Cell lysates were harvested and then purified with an AKTA purifier (GE Healthcare, Zurich, Switzerland). Three IGFBP7 fragments were constructed: N (1–171), L (172–244) and C (245–282). Four IGFBP7 mutants were constructed: Arg198-Glu, Arg198-Ile and His200-Phe, Arg198-Ile, and His200-Phe.

### Western blot analysis

20 μl of insulin (1 μg/μl) was subjected to SDS–PAGE (8% or 12% gels) and then electroblotted on to nitrocellulose membranes to detect the binding ability of IGFBP7 fragments to insulin. The membranes were washed with Tris-buffered saline-0.1% Tween 20 (TBS-T) and then incubated for 1 h at room temperature with a 1:3000 dilution of IGFBP7 wild-type or fragments for 2 h. After washing with TBS-T, the membranes were incubated with anti-His antibody and then incubated in fluorescence-labeled anti-mouse IgG secondary antibodies (Amersham Pharmacia Biotech, Piscataway, NJ). The bands were detected with the Odyssey infrared imaging system and quantified using an image analyzer (GS-700) equipped in the system.

### Dot blot analysis

2.5 μl of the sample with the same concentration was dotted directly on to nitrocellulose membranes. The membranes were blocked for 1 h at room temperature in 1% bovine serum albumin (BSA)/TBS-T and then incubated at 4 °C overnight with 1 μg/ml insulin. The membranes were washed with TBS-T and then incubated for 1 h at room temperature with a 1:3000 dilution of insulin antibody for 2 h. After washing with TBS-T, the membranes were incubated in fluorescence-labeled anti-rabbit IgG secondary antibodies (Amersham Pharmacia Biotech, Piscataway, NJ). The bands were detected with the Odyssey infrared imaging system as described above. As controls, the membranes were incubated in IGFBP7 monoclonal antibody, washed, and then incubated with fluorescence-labeled anti-mouse IgG secondary antibodies.

### Pull-down Assay

Pull-down assay was performed on an Äkta Explorer (GE Healthcare, Zurich, Switzerland). His6-taged-IGFBP7 (0.1 mg, wild-type and mutants) were mixed with 0.5 mg insulin. The mixture was loaded on to a Ni-Sepharose 6 column (GE Healthcare). The column was washed with 20 mM Tris-HCl, pH 8.0, 150 mM NaCl, and 20 mM imidazole, and His6-tagged protein was eluted with elution buffer (the same as the binding buffer except for 250 mM imidazole). The eluted materials were subjected to SDS–PAGE and western blot with anti-IGFBP7 or anti-insulin antibody. Pull-down experiments were performed at 4 °C.

### Computational methods

MD/SMD simulations, free-energy calculations, and electrostatic potential calculations were performed to predict the interaction sites and identify the hot-spot residues.

All simulations were performed with GROMACS 4.6.5^39^,^40^,^41^ using Charmm27 All-Atoms (AA) force field^42^ in the NpT ensemble. The Nose-Hoover thermostat and Parrinello-Rahman pressure coupling were applied to the system to maintain a constant temperature of 25 °C and a constant pressure of 101.3 kPa. Simulations were carried out with a time step of 2fs. The cut-off of non-bonded van der Waals interaction with a switching function started at a distance of 12 Å and reached zero at 13.5 Å (Note that in this section, we are retaining the use of Å, although we recognize that generally this has been superseded as a scientific unit by nanometer). The particle mesh Ewald summation^43^ was used to calculate the long-range electrostatic interactions with a cut-off distance of 12 Å for the separation of the direct and reciprocal space. Proteins were solvated in simple point charge water molecules forming a box. Sodium and chloride ions were added to electrically neutralize the system of 0.15 M NaCl. Periodic boundary conditions were used in all simulations and bond lengths were constrained by the linear constraint solver algorithm^44^. Each system conducted a 2 ns MD simulation for pre-equilibrium. Constant velocity pulling is a basic method of SMD. The constant velocity v was fixed at 1 nm ns^−1^ and the spring constant k was set to 30000 kJ mol^−1^nm^−2^ to obtain the best fit of SMD observation window and the interaction energy curves. As for free-energy calculations, umbrella sampling method was used to obtain the potential of mean force (PMF) profile. The force constant was set at 2000 kJ mol^−1^. 135 umbrella sampling windows were chosen for H200F system to make a good and reasonable overlap between windows for calculating the PMF while the numbers of other two mutants R198I, R198I with H200F are 183 and 159, respectively. The simulation time of each window was set to 3 nanoseconds. The free energy profiles and error bars were calculated with weighted histogram analysis method and bootstrap analysis method^45^,^46^.

## Additional Information

**How to cite this article**: Ruan, W. *et al.* Interaction between IGFBP7 and insulin: a theoretical and experimental study. *Sci. Rep.* **6**, 19586; doi: 10.1038/srep19586 (2016).

## Supplementary Material

Supplementary Information

## Figures and Tables

**Table 1:** Qualitative strength of interactions of IGFBP7 mutants and insulin.

Number	Mutants	Strength of interactionbetween IGFBP7 andinsulin
1	Wild-type	Strong
2	His200Phe	Strong
3	Arg198Ile	Strong
4	Arg198Glu	Weak
5	Arg198Glu His200Phe	Weak
