# Supplementary material for: Interaction between IGFBP7 and insulin: a theoretical and experimental study
Source: Sci Rep. 2016 Apr 22;6:19586. doi: 10.1038/srep19586 (PMC4840315; doi:10.1038/srep19586)
Supplement: Supplementary Information [file srep19586-s1.doc]

**Supplementary information**

**Interaction between IGFBP7 and insulin: a theoretical and experimental *study***

*Wenjing Ruan^ac,1^, Zhengzhong Kang^b,1^, Youzhao Li^a^, Tianyang Sun^b^, Lipei Wang^a^, Lijun Liang^b^, Maode Lai^a,*^, Tao Wu^b,*^*

the imidazole side chain of histidine plays an important role in protein function. The pKa of the histidine side chain is close to physiological pH which allows it to act as an acid or a base. For histidine residue, the deeper it buried within the protein, the lower pKa value it will have.[^1^](#_ENREF_1) Compared with other histidine residues, H200 has the highest pKa predicted by PROPKA since it is located on the surface of the protein and contact with insulin. In native state, the imidazole ring of histidine normally exist in neutral states while in intermediate state when folding or reacting with other proteins, the side chain has a large distributions in protonated state.[^2^](#_ENREF_2) So here it will be more reasonable for H200 to be in charged state when it reacts with insulin. The structure is shown below:


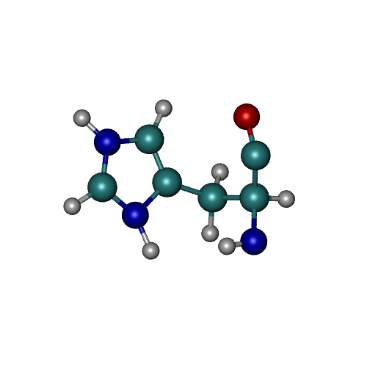


Figure 1. Protonated state of Histidine. Deep blue color represents the nitrogen atoms while light blue color for carbon atoms and white color for hydrogen atoms with red color for oxygen atoms. Each nitrogen atom in the ending part is bonded to one hydrogen bond. The carbon atom between these two nitrogen atoms share the same bonds with neighbor nitrogen atoms.

1 Edgcomb, S. P. & Murphy, K. P. Variability in the pKa of histidine side-chains correlates with burial within proteins. *Proteins* **49**, 1-6, doi:10.1002/prot.10177 (2002).

2 Hansen, A. L. & Kay, L. E. Measurement of histidine pKa values and tautomer populations in invisible protein states. *Proc Natl Acad Sci U S A* **111**, E1705-1712, doi:10.1073/pnas.1400577111 (2014).
